# Inequities in Community Exposure to Deadly Gun Violence by Race/Ethnicity, Poverty, and Neighborhood Disadvantage among Youth in Large US Cities

**DOI:** 10.1007/s11524-022-00656-0

**Published:** 2022-06-07

**Authors:** Nicole Kravitz-Wirtz, Angela Bruns, Amanda J. Aubel, Xiaoya Zhang, Shani A. Buggs

**Affiliations:** 1 Violence Prevention Research Program, Department of Emergency Medicine, University of California Davis School of Medicine, 2315 Stockton Boulevard, Sacramento, CA 95817 USA; 2 Department of Sociology & Criminology, Gonzaga University, Spokane, WA USA; 3 Department of Human Ecology, University of California Davis, Davis, CA USA

**Keywords:** Gun violence, Community violence, Race-ethnicity, Poverty, Neighborhood disadvantage, Youth

## Abstract

Understanding the burden of gun violence among youth is a public health imperative. While most estimates are based on direct and witnessed victimization, living nearby gun violence incidents may be consequential too. Yet detailed information about these broader experiences of violence is lacking. We use data on a population-based cohort of youth merged with incident-level data on deadly gun violence to assess the prevalence and intensity of community exposure to gun homicides across cross-classified categories of exposure distance and recency, overall and by race/ethnicity, household poverty, and neighborhood disadvantage. In total, 2–18% of youth resided within 600 m of a gun homicide occurring in the past 14–365 days. These percentages were 3–25% for incidents within 800 m and 5–37% for those within a 1300-m radius. Black and Latinx youth were 3–7 times more likely, depending on the exposure radius, to experience a past-year gun homicide than white youth and on average experienced incidents more recently and closer to home. Household poverty contributed to exposure inequities, but disproportionate residence in disadvantaged neighborhoods was especially consequential: for all racial/ethnic groups, the difference in the probability of exposure between youth in low vs high poverty households was approximately 5–10 percentage points, while the difference between youth residing in low vs high disadvantage neighborhoods was approximately 50 percentage points. Given well-documented consequences of gun violence exposure on health, these more comprehensive estimates underscore the importance of supportive strategies not only for individual victims but entire communities in the aftermath of gun violence.

Firearm homicide is consistently a leading cause of death among children and teens ages 0–19 in the USA, accounting for more than 2800 deaths (8/day) in 2020 [1]. But the burden of community gun violence, and particularly gun homicide, is not distributed evenly; rather, these patterns reflect a constellation of mutually constitutive inequities in the macro-level systems and social, economic, and political forces that shape the conditions of daily life, with Black and Latinx youth disproportionately exposed to communities experiencing high risk factors associated with violence and low protective factors associated with safety [2, 3]. Consequently, average rates of gun homicide for 2016–2020 are more than 13 times and 2 times higher among Black (10.6/100,000) and Latinx (1.9/100,000) children and teens, respectively, compared with that of white peers (0.8/100,000) [1].

Yet deaths due to firearm assault represent only a fraction of the human toll of the community gun violence problem. Nonfatal firearm injuries are estimated to outnumber firearm homicides by more than 2 to 1 [4]. Indirect exposure to gun violence in the form of hearing gunshots or witnessing gunfire is also prevalent. Nationally, in 2013–2014, an estimated 13% of adolescents ages 14–17 reported having heard gunshots or having seen someone shot over their lifetime [5]. One study among communities highly impacted by structural and interpersonal violence found 56% of youth ages 12–15 in 1997–2000 reported having heard gunshots in the past year [5]. Violent victimization and indirect exposure to violence have been associated with a wide range of detrimental socioemotional and health risk outcomes, particularly when firearms are involved, as well as subsequent self- and other-directed violence perpetration [7–14].

While the prevalence and consequences of direct and indirect exposure to violence have been increasingly well documented, there is growing recognition that conceptions of exposure and resulting trauma can extend beyond the individual and affect the entire community [15, 16]. This expanded view of “community exposure to violence” encompasses not only the typical areas of study in past research, such as causing or being the victim of harm and hearing or seeing violence firsthand, but also spillover effects that result from social proximity to individuals and communities that are disproportionately affected by violence, as well as living or spending time in places impacted by elevated rates of violence, regardless of whether the violence is witnessed or experienced directly.

Empirical support for this expanded view of exposure is growing. For example, ethnographic research in violence-impacted neighborhoods suggests that youth frequently navigate strategically through public spaces, shifting their schedules, their networks, and their routines in response to community violence [17–20]. Quasi-experimental studies conducted in New York City and Chicago have documented declines in cognitive functioning, lower levels of attention and impulse control, and worse standardized test performance among children in the aftermath of a homicide that occurred near their home relative to children who resided in the same neighborhood but who were assessed at a time when no violence had occurred [21–24]. A recent study in Philadelphia found an increase in children’s mental health-related emergency department utilization in the 2 months following the occurrence of a shooting within 2–3 blocks of their home [25].

Most scholarship that has adopted this broader perspective on community exposure to violence has relied on small or geographically restricted populations, due in part to the absence of a harmonized national database of violent incidents in the USA [26]. A handful of studies using population-based samples of children and families have increased the knowledge base on exposure to violence in broader community context and the generalizability of its consequences: one study found racial-ethnic and income inequalities in past-year exposure to deadly gun violence occurring within 500 m of youths’ homes and schools [27], and two studies have documented negative impacts of these exposures on youths’ mental health and behavioral outcomes [28, 29]. However, detailed information on the distribution and determinants of this problem is still lacking. Specifically, there is a paucity of studies of community exposure disparities at the nexus of not only individual-level race-ethnicity and income, as examined in past research, but also neighborhood characteristics that contribute to community violence and safety. The omission of place-based factors is a notable gap in the literature given the persistence of racial-ethnic inequities in access to well-resourced and salutatory residential environments across all levels of income [30].

The current study extends our understanding of the interrelationships among these social-ecological determinants and community exposure to violence using a unique combination of nationwide population-based, individual-level data on youth, their families, and neighborhoods merged with incident-level data on deadly gun violence. Specifically, we report estimates of the prevalence of community exposure to gun homicides across cross-classified categories of exposure distance and recency, overall and by individual, household, and neighborhood-level demographic and socioeconomic markers of structural inequity, among a representative cohort of youth in large US cities. Descriptive data with this level of detail have not, to our knowledge, been published previously and can provide a critical foundation on which to base future research, as well as place-based violence prevention, intervention, and supportive strategies that better reflect the spatial and temporal ripple effects of firearm violence and trauma among youth and families in communities as a whole, beyond direct and witnessed victimization [31].

## Methods

### Data

We used individual-level data on youth and their families from the sixth wave of the Fragile Families and Child Wellbeing Study (FFCWS), a birth cohort study following a stratified, multistage, probability sample of 4898 children born 1998–2000 in US cities with populations in excess of 200,000 [32]. Interviews were conducted at the focal child’s birth and subsequently at ages 1, 3, 5, 9, and 15, spanning years 1999–2017. For the sixth wave of interviews, when children were approximately 15 years of age (hereafter, Year 15), the completion rate among baseline sample members was 73% for primary caregivers (*N* = 3580) and 70% for youths themselves (*N* = 3444), which is comparable to or higher than response rates for other household panel surveys [33]. The analytic sample for this study includes the 2471 youth in the 16-city national sample (the FFCWS also includes respondents in four cities that were not part of the stratified random sample) who completed the Year 15 interview and for whom the latitude and longitude coordinates of their home address were known.

Incident-level data on gun violence came from the Gun Violence Archive (GVA), a national open-source database of incidents of gun violence and gun crime for years 2014 onward. Incidents are identified by professional staff on a daily basis through automated queries and manual research through over 7500 sources from local and state police, media, data aggregates, government, and other resources, providing near real-time data [34]. A prior analysis comparing 2014–2016 GVA data to mortality statistics from the Centers for Disease Control and Prevention (CDC) found a strong correlation (*r* = 0.97), with secular changes and seasonal trends reflected clearly in both sources [35]. GVA data on the date and location of deadly gun violence incidents (including murder-suicides, but not suicides) were linked by FFCWS staff to the FFCWS Year 15 interviews using the latitude and longitude coordinates of youths’ home addresses and the dates of their interviews. Access to the merged FFCWS-GVA file was provided under a restricted use data contract between the authors and the Center for Research on Child Wellbeing at Princeton University.

### Measures

#### Community Exposure to Gun Homicides

We characterized the prevalence and intensity (or dose: number of incidents) of community exposure to gun homicides^1^ across cross-classified categories of distance and recency as follows: living within 600, 800, and 1300 m of an incident in which an individual was killed with a firearm as a result of interpersonal violence in the 14-, 30-, and 365-day periods before youth were interviewed at Year 15. Distance measures correspond to the approximate radius of census block groups (600 m), subjective perceptions of a typical neighborhood (800 m), and census tracts (1300 m) [36], and the time periods are consistent with those used in prior research of the effects of community violence exposure on social and health outcomes [21, 27, 28]. Because some youth (19% of the study sample) were interviewed in 2014 when less than 1 full year of GVA data were available, we do not include them in our estimates of past-year exposure.

#### Social-Ecological Markers of Structural Inequity

At the individual level, as a marker of structural inequities resulting from racist policies, we utilized a FFCWS-constructed variable for youth race-ethnicity, which was based on their self-reported status and then recoded according to census-recognized categories and collapsed to maximize within-group sample sizes: non-Hispanic Black; Hispanic/Latino; non-Hispanic white; non-Hispanic other, including American Indian or Alaska Native, Asian, and Native Hawaiian or Pacific Islander; and multi-racial (hereafter, Black, Latinx, white, and other youth of color, encompassing both non-Hispanic other and multi-racial youth). Mothers’ self-reported race-ethnicity was substituted when youths’ own reports of race-ethnicity were unknown or missing.

Household poverty status was measured as the ratio of total household income to the prior year poverty thresholds established by the US Census Bureau and categorized as follows, consistent with past research [27]: low (200% or more of the poverty threshold), moderate (100–199% of the poverty threshold), and high poverty (less than 100% of the poverty threshold).

Neighborhood socioeconomic disadvantage was constructed from a principal component analysis of six standard census tract-level items [37, 38]: rates of (1) poverty, (2) unemployment, (3) households that are female-headed, and (4) public assistance receipt, along with the percentages of persons age 25 and older who (5) lacked a high school diploma and (6) held a college degree. Scores on this composite index of neighborhood disadvantage were divided into tertiles (low, moderate, and high disadvantage) based on the distribution in the study sample.

Sample characteristics by race-ethnicity, household poverty status, and neighborhood disadvantage, along with other sociodemographic factors, are provided in Table 1.

**Table 1:** Sociodemographic characteristics of the analytic sample of youth, Fragile Families and Child Wellbeing Study, 2014–2017 (*N* = 2471)

Characteristic	Weighted % (95% *CI*)
**Age (years)**	
14	0.13% (0.00–11.55)
15	78.02% (67.11–86.06)
16	20.03% (12.84–29.86)
17	1.67% (1.05–2.65)
18	0.13% (0.06–0.31)
**Sex**	
Male	55.99% (49.70–62.11)
Female	44.01% (37.89–50.31)
**Race-ethnicity**	
Black	23.99% (17.71–31.65)
Latinx	30.74% (22.87–39.92)
White	35.52% (30.74–40.61)
Other/multi-racial	9.75% (6.99–13.45)
**Primary caregiver**	
Biological mother	90.56% (87.47–92.95)
Biological father	7.15% (4.76–10.61)
Non-parental caregiver	2.29% (1.68–3.12)
**Living arrangement**	
Biological mother and father	43.12% (39.10–47.24)
Biological mother and her new partner	20.40% (16.69–24.70)
Biological mother only	27.03% (22.46–32.15)
Biological father and his new partner	3.28% (1.55–6.78)
Biological father only	3.88% (2.25–6.60)
Other primary caregiver	2.29% (1.68–3.12)
**Education of primary caregiver**	
< High school	14.89% (6.20–31.68)
High school or equivalent	18.32% (14.33–23.12)
Some college/technical school	36.27% (29.19–44.00)
College or graduate degree	29.82% (21.49–39.75)
**Primary caregiver currently employed**	
Yes	75.05% (69.76–79.69)
No	24.73% (20.16–29.96)
**Household size (mean/SE)**	4.61 (0.12)
**No. of kids in household (mean/SE)**	2.45 (0.67)
**Household poverty status**	
Low (200% + FPL)	55.27% (46.85–63.39)
Moderate (100–199% FPL)	21.75% (16.54–28.05)
High (< 100% FPL)	22.58% (17.59–28.48)
**Housing arrangement of primary caregiver**	
Rent	42.37% (38.16–46.69)
Live w/ family/friends and pay rent	2.46% (0.85–6.90)
Live w/ family/friends and don’t pay rent	1.93% (1.00–3.68)
Own home	48.21% (42.11–54.36)
Live in home owned by family	3.30% (1.99–5.41)
Live in temporary housing/group shelter	0.64% (0.22–1.82)
Other	0.92% (0.48–1.76)
Home in public housing project*	
Yes	13.03% (9.53–17.58)
No	86.42% (81.77–90.02)
**Primary caregiver moved since last interview**	
Yes	48.73% (43.53–53.95)
No	51.04% (45.85–56.20)
**Neighborhood disadvantage**	
Low	41.94% (36.39–47.70)
Moderate	33.77% (28.96–38.93)
High	24.13% (20.73–27.89)
**US region**	
West	5.78% (2.53–12.70)
Midwest	12.34% (8.73–17.15)
South	51.14% (46.76–55.49)
East	30.62% (26.53–35.05)
Noncontiguous	0.12% (0.03–0.51)

^*^Not asked of primary caregivers who own their own home or those who are living in temporary housing/group shelter (*N* = 1571)

Note: columns may not sum to 100% due to don’t knows and nonresponse

### Statistical Analysis

We calculated weighted percentages and their corresponding 95% confidence intervals (*CI*) of youth with community exposure to gun homicide incidents at cross-classified categories of exposure distance and recency, overall and by categories of race-ethnicity, poverty, and neighborhood disadvantage, using standard descriptive techniques in Stata, version 15.1 (StataCorp LP, College Station, TX, USA). For past-year exposure, we delineated exposure intensity (or dose), across various distances, as the number of incidents: 1, 2, or 3 or more. These counts were determined to allow for comparable numbers of youth across categories of intensity and based on prior research documenting a dose-responsive relationship between exposure to childhood adversity and future social and health problems [39–42]. Mean distance and time, and their corresponding standard errors (SE), to the nearest and most recent incident of deadly gun violence, respectively, were also computed. We employed weighted logistic regression to assess the nexus of community exposure disparities and race-ethnicity, household poverty status, and neighborhood disadvantage, which were included in this model simultaneously. Predicted probabilities of gun homicide exposure are presented to facilitate interpretation. Sampling weights were incorporated throughout using the SVY and weighting commands to adjust for the FFCWS sample design (probability of selection), non-response at baseline, and attrition based on observed characteristics over the waves. Weighted estimates are designed to be statistically representative of youth born in large US cities between 1998 and 2000.

This study was deemed exempt from human subjects review by the Institutional Review Boards at the University of California Davis and Gonzaga University because only secondary, deidentified data were used.

## Results

### Exposure Prevalence: by Distance and Recency

 More than 1 in 3 youth (37.2%; 95% *CI* = 32.7–41.9) experienced at least 1 gun homicide within 1300 m of their home in the past year, 8.5% (95% *CI* = 6.3–11.5) in the past 30 days, and 5.1% (95% *CI* = 3.0–8.6) in the past 2 weeks (Table 2). Thirteen percent of youth (13.1%; 95% *CI* = 10.0–17.0) experienced 3 or more incidents within 1300 m of home in the past year. One-quarter of youth (25.1%; 95% *CI* = 21.2–29.4) lived within 800 m, or approximately half a mile, of a gun homicide occurring in the past year; 6.5% (95% *CI* = 4.4–9.5) experienced 3 or more such incidents. At 600 m from home, corresponding to the median radius of a census block group, the prevalence of past-year exposure to gun homicides was 18.0% (95% *CI* = 15.1–21.3); 2.7% (95% *CI* = 1.5–5.0) of youth experienced 3 or more such incidents.

**Table 2:** Percentage of youth exposed to deadly gun violence incidents, by distance and recency, Fragile Families and Child Wellbeing Study, 2014–2017 (*N* = 2471)*

	Unweighted *N*	Weighted % (95% *CI*)	Est. population size
**1300 m**			
14 days	120	5.09% (2.97–8.58)	56,502
30 days	272	8.52% (6.26–11.49)	94,574
365 days	871	37.19% (32.72–41.88)	398,141
1	335	17.17% (13.50–21.59)	183,807
2	162	6.93% (5.13–9.30)	74,181
3 +	374	13.09% (9.99–16.97)	140,153
**800 m**			
14 days	58	3.15% (1.34–7.14)	34,963
30 days	136	4.93% (2.91–8.24)	54,765
365 days	599	25.08% (21.2–29.4)	268,495
1	295	13.42% (10.24–17.41)	143,703
2	132	5.17% (3.80–6.99)	55,313
3 +	172	6.49% (4.37–9.53)	69,478
**600 m**			
14 days	38	2.00% (0.75–5.23)	22,218
30 days	88	3.39% (1.88–6.05)	37,645
365 days	454	18.00% (15.13–21.26)	192,671
1	260	11.72% (9.48–14.41)	125,499
2	89	3.56% (2.20–5.69)	38,069
3 +	105	2.72% (1.46–5.00)	29,103

^*^Because some youth (19%) were interviewed in 2014 when less than 1 full year of GVA data were available, we do not include those youth in the past-year estimates (*N* = 1991)

Among youth who experienced a gun homicide in the past year, the nearest incident occurred, on average, 750.7 m (*SE* = 30.7) from their home (Table 3). For those who experienced a gun homicide in the past month and the past 2 weeks, the nearest incident occurred, on average, 892.7 m (*SE* = 60.9) and 863.4 m (*SE* = 101.7) from home, respectively. Similarly, of youth who experienced a gun homicide within 1300, 800, and 600 m of their home, the most recent incident occurred, on average, in the past 123.5 days (*SE* = 8.6), 128.5 days (*SE* = 10.8), and 135.5 days (*SE* = 12.3), respectively.

**Table 3:** Average time and distance to the most recent and nearest deadly gun violence incident, respectively, among youth exposed to violence, by distance and recency, Fragile Families and Child Wellbeing Study, 2014–2017*

	Mean	*SE*	95% *CI*
**Time to most recent incident (days)**			
600 m	135.46	12.32	110.36–160.55
800 m	128.52	10.79	106.56–150.49
1300 m	123.50	8.61	105.95–141.04
**Distance to nearest incident (meters)**			
14 days	863.43	101.71	656.26–1070.60
30 days	892.74	60.89	768.71–1016.78
365 days	750.70	30.74	688.09–813.30

^*^Sample sizes vary by distance and recency categories; *N* = 536 (600 m); *N* = 715 (800 m); *N* = 1031 (1300 m); *N* = 166 (14 days); *N* = 340 (30 days); *N* = 982 (365 days, excluding youth who were interviewed in 2014 when less than 1 full year of GVA data were available)

### Exposure Inequities: Nexus of Race-Ethnicity, Poverty, and Neighborhood Disadvantage

**Fig. 1:**
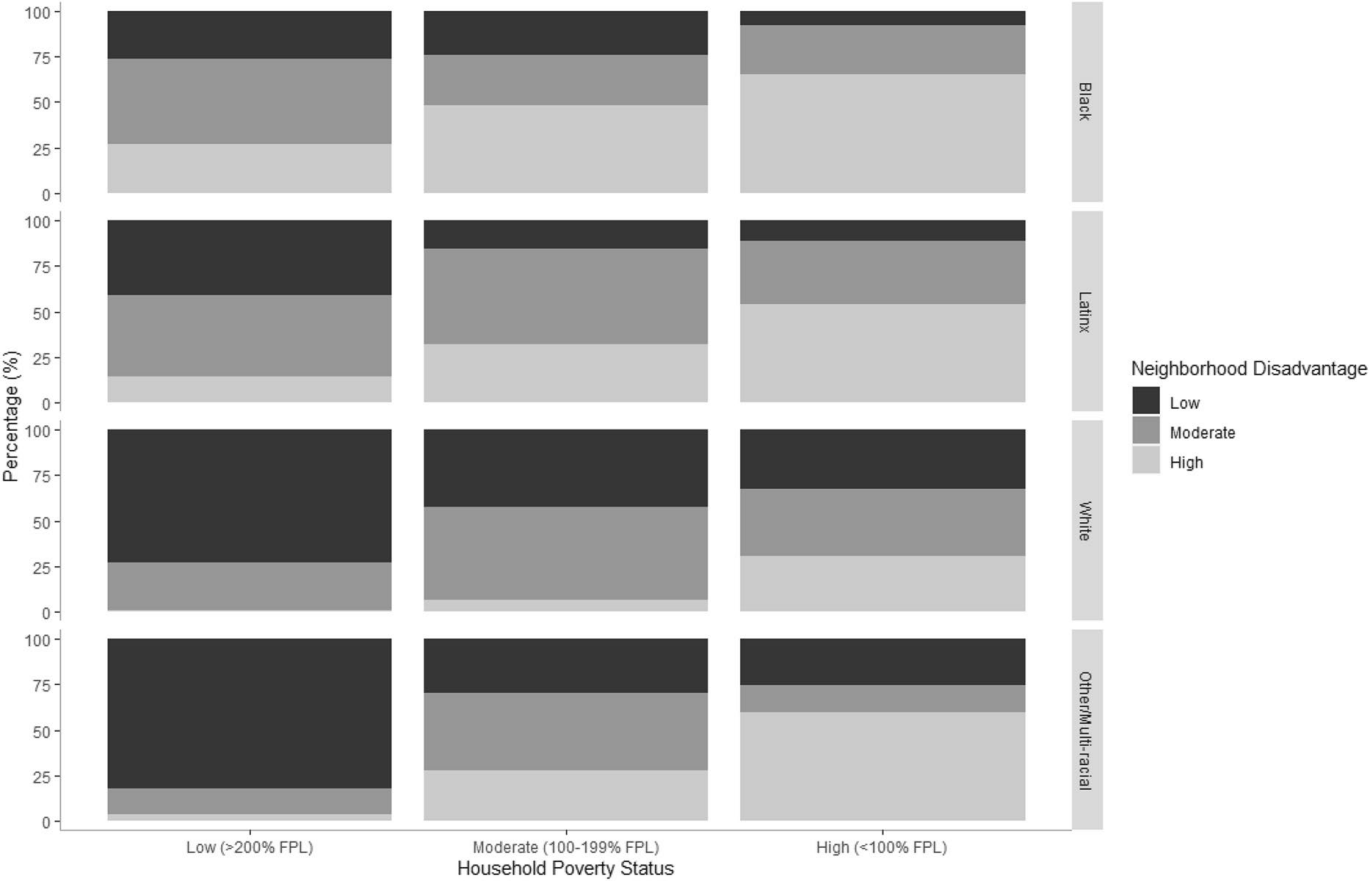
Distribution of youth across cross-classified categories of household poverty status and neighborhood disadvantage, by race-ethnicity, Fragile Families and Child Wellbeing Study, 2014–2017 (*N* = 2471)

Black, Latinx, and other youth of color occupied distinctive residential environments relative to white youth at every level of household income. Figure 1 and Table 6 in the Appendix depict the distribution of youth by race-ethnicity across cross-classified categories of household poverty status and neighborhood socioeconomic disadvantage. As shown, nearly 3 in 4 Black youth in low-poverty households resided in moderate (47.3%; 95% *CI* = 33.7 − 61.2) or high (26.7%; 95% *CI* = 16.1 − 40.8) disadvantage neighborhoods, compared with only 1 in 4 white youth (25.7% [95% *CI* = 16.5 − 37.7] in moderate and 0.9% [95% *CI* = 0.1 − 7.0] in high disadvantage neighborhoods). Conversely, the majority (65.1%; 95% *CI* = 55.7 − 73.5) of Black youth in high-poverty households resided in high-disadvantage neighborhoods, whereas white youth in high-poverty households were more evenly distributed across high, moderate, and low-disadvantage neighborhoods (30.7%, 36.4%, and 32.8%, respectively). This pattern was mostly similar but less pronounced for Latinx and other youth of color compared with white youth.

Community exposure to gun homicides, in turn, was a more frequent experience in the lives of Black and Latinx youth than white peers (Table 4). Fifty-six percent of Black youth (56.3%; 95% *CI* = 48.1 − 64.2) and nearly half of Latinx youth (48.6%; 95% *CI* = 35.7 − 61.7) lived within 1300 m of a gun homicide in the past year; 1 in 4 Black youth (26.0%; 95% *CI* = 18.8 − 34.8) and 1 in 5 Latinx youth (19.2%; 95% *CI* = 10.5 − 32.6) experienced 3 or more incidents. The rate for white youth was 16.8% (95% *CI* = 9.9 − 27.0) for any incident and less than 1% in the case of 3 or more incidents (0.6%; 95% *CI* = 0.1 − 2.2). Of those who experienced a gun homicide within 1300 m in the past year, Black and Latinx youth were also more likely to live in closer proximity to the nearest incident (mean [*SE*] = 682.0 [41.6] m and 712.3 [48.5] m, respectively) and for the last incident to have occurred more recently (mean [*SE*] = 116.6 [9.6] days and 109.2 [16.0] days, respectively) compared with white youth (mean [*SD*] = 989.1 [71.1] m and 148.4 [11.2] days) (Table 5). A similar pattern of results was evident across all cross-classifications of exposure distance and recency.

**Table 4:** Percentage of youth exposed to deadly gun violence incidents, by distance, recency, and race-ethnicity, Fragile Families and Child Wellbeing Study, 2014–2017 (*N* = 2471)*

	Weighted % (95% *CI*)			
	Black	Latinx	White	Other/multi
**600 m**				
14 days	1.31% (0.44–3.84)	5.40% (1.48–17.77)	0.03% (0.00–0.16)	0.19% (0.04–0.89)
30 days	4.05% (2.17–7.42)	6.47% (2.52–15.62)	0.68% (0.15–3.08)	1.94% (0.35–10.04)
365 days	28.22% (21.80–35.68)	26.61% (18.63–36.48)	3.68% (1.87–7.12)	18.64% (7.32–39.93)
1	16.98% (10.81–25.64)	16.75% (11.45–23.85)	2.65% (1.10–6.21)	16.48% (5.34–40.85)
2	6.72% (3.72–11.84)	4.58% (1.49–13.26)	1.02% (0.28–3.63)	1.94% (0.20–16.22)
3 +	4.53% (2.83–7.18)	5.28% (1.82–14.38)	0.01% (0.00–0.12)	0.21% (0.04–1.07)
**800 m**				
14 days	5.45% (1.65–16.55)	5.90% (1.75–18.06)	0.03% (0.00–0.16)	0.19% (0.04–0.89)
30 days	8.33% (3.86–17.06)	7.58% (3.12–17.33)	0.70% (0.16–3.05)	3.64% (0.95–13.01)
365 days	37.88% (32.42–43.67)	35.42% (24.25–48.43)	7.57% (4.12–13.50)	25.77% (12.10–46.69)
1	15.69% (11.22–21.51)	17.66% (10.94–27.24)	6.53% (3.13–13.14)	19.89% (7.38–43.61)
2	10.56% (7.45–14.75)	6.17% (2.91–12.60)	1.02% (0.28–3.63)	4.15% (1.34–12.13)
3 +	11.64% (6.56–19.81)	11.59% (5.67–22.23)	0.01% (0.00–0.12)	1.73% (0.12–21.25)
**1300 m**				
14 days	9.38% (4.42–18.80)	7.79% (3.43–16.77)	0.67% (0.16–2.67)	2.09% (0.43–9.68)
30 days	16.27% (10.76–23.85)	11.22% (6.33–19.13)	1.69% (0.70–4.02)	5.77% (1.88–16.38)
365 days	56.34% (48.12–64.22)	48.60% (35.72–61.66)	16.81% (9.95–26.99)	29.61% (14.71–50.66)
1	18.57% (12.69–26.36)	21.41% (15.04–29.54)	13.17% (7.58–21.91)	15.08% (4.52–40.01)
2	11.79% (7.97–17.12)	7.95% (3.94–15.39)	3.07% (1.24–7.43)	6.07% (1.33–23.69)
3 +	25.97% (18.75–34.77)	19.24% (10.52–32.56)	0.57% (0.14–2.25)	8.47% (3.54–18.90)

^*^Because some youth (19%) were interviewed in 2014 when less than 1 full year of GVA data were available, we do not include those youth in the past-year estimates (*N* = 1991)

**Table 5:** Average time and distance to the most recent or nearest deadly gun violence incident, respectively, among youth exposed to violence, by distance, recency, and race-ethnicity, Fragile Families and Child Wellbeing Study, 2014–2017*

	Mean (SE)			
	Black	Latinx	White	Other/Multi-racial
**Time to most recent incident (days)**				
600 m	142.09 (9.69)	100.80 (16.86)	181.72 (42.46)	230.33 (88.32)
800 m	134.64 (15.97)	94.78 (14.58)	188.78 (25.22)	185.97 (74.78)
1300 m	116.61 (9.62)	109.19 (16.03)	148.42 (11.24)	177.38 (64.16)
**Distance to nearest incident (meters)**				
14 days	941.02 (95.09)	732.41 (170.78)	949.91 (176.05)	1248.69 (205.38)
30 days	951.15 (46.78)	762.99 (108.23)	1087.86 (206.71)	931.27 (270.47)
365 days	681.96 (41.57)	712.29 (48.49)	989.12 (71.13)	652.37 (67.62)

^*^Sample sizes vary by distance and recency categories; *N* = 536 (600 m); *N* = 715 (800 m); *N* = 1031 (1300 m); *N* = 166 (14 days); *N* = 340 (30 days); *N* = 982 (365 days, excluding youth who were interviewed in 2014 when less than 1 full year of GVA data were available)

**Fig. 2:**
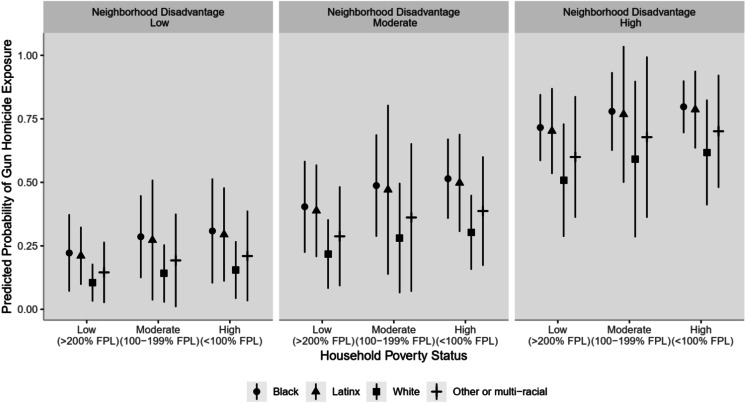
Predicted probability of youths’ exposure to gun homicide incidents in the past year within 1300 m of home by race-ethnicity, household poverty status, and neighborhood disadvantage, Fragile Families and Child Wellbeing Study, 2014–2017 (*N* = 1991)

Results from the weighted logistic regression model document the compounding impacts of household-level and neighborhood-level deprivation on the predicted prevalence of past-year community exposure to gun homicides within 1300 m of home across categories of race-ethnicity, underscoring the relatively more prominent role of neighborhood factors in shaping exposure risk (Fig. 2 and Table 7 in the Appendix). Specifically, for all racial-ethnic groups, at every level of neighborhood socioeconomic disadvantage, adjusting the poverty level of youths’ households from high to relatively low poverty (holding neighborhood disadvantage constant) reduced their chances of community exposure to gun homicides by approximately 5 − 10 percentage points. On the other hand, at every level of household poverty, adjusting instead the level of disadvantage in youths’ neighborhoods from high to low disadvantage (holding household poverty constant) reduced their chances of community exposure to gun homicides by approximately 50 percentage points.

## Discussion

The USA suffers from uniquely high rates of gun violence compared with other similarly wealthy countries, and direct or witnessed victimization is only the tip of the iceberg when it comes to capturing the full scope of this problem in daily life [15]. In this study examining a cohort of children born in large US cities between 1998 and 2000 and followed up at approximately 15 years of age, community exposure to gun violence—specifically, exposure that results from living or spending time in places highly impacted by gun violence, regardless of whether the violence is witnessed or experienced firsthand—was a frequent experience in the lives of youth, particularly Black and Latinx youth. For these youth, exposure reflected, in part, disproportionate residence in localities characterized by the social and economic consequences of long-standing structural disinvestment and institutional racism that have contributed to the conditions in which community gun violence is more likely to emerge.

Prior national surveys indicate that less than 5% of 14 − 17-year-olds have witnessed a shooting in the past year [5]; yet our findings, in which exposure to violence is conceptualized in broader community context, suggest that more than 1 in 3 youth (37%) reside in localities (characterized by less than a 1-mile radius) that have experienced at least 1 gun homicide in the prior year; more than 1 in 10 youth (13%) have experienced 3 or more such incidents. Among Black and Latinx youth, respectively, these percentages were 56% and 49% for exposure to 1 or more gun homicides and 26% and 19% for 3 or more incidents—roughly 3 times the rate of white youth for any incident and 20 times the rate for multiple incidents. These collective experiences of community violence can be consequential too.

As noted in our introduction, a growing body of research has found that the occurrence of a violent incident near a child’s home is associated with subsequent school and behavioral problems, as well as acute mental health-related emergency department utilization [21–25, 28]. There is additional evidence to suggest a graded dose–response relationship between community violence exposure and adverse outcomes, including increasing levels of posttraumatic stress symptoms and delinquent behaviors [43]. Until recently, however, constraints on the availability of incident-level violence data have limited our understanding of the extent to and ways in which youth are touched by gun violence beyond witnessing or experiencing it directly. To our knowledge, this study provides the most detailed information on the prevalence of such broader community exposure to deadly gun violence among a population-based sample of US youth.

Notably, community gun violence exhibits a high degree of microspatial concentration [44, 45]. In some cities, roughly 5% of city blocks account for as much as 50% of gun violence [46]. Consistent with other work on racial residential segregation and neighborhood stratification in the USA [47], our findings indicate that Black and, to a lesser extent, Latinx youth reside more often in socioeconomically disadvantaged areas, e.g., the largest percentage of all Black youth in our sample resided in high-poverty households in high-disadvantage areas (21%) while the majority of all white youth resided in low-poverty households in low-disadvantage areas (58%; Table 8 in the Appendix). Prior research has found that racialized economic segregation in residential places, an enduring impact of 1930s-era redlining and other historical and current-day practices of racialized housing discrimination, increases the likelihood of firearm violence [48–51]. Our results underscore the compounding effects of household poverty and neighborhood socioeconomic disadvantage on community violence exposure among youth, with the chance of past-year exposure to gun homicides ranging from 10 to 22%, depending on racial-ethnic subgroup, among youth who live in low-poverty households in low-disadvantage localities to as high as 62–80% for those in high-poverty households in high-disadvantage localities.

Our analyses examining the nexus of individual race-ethnicity, household poverty, and neighborhood socioeconomic disadvantage yielded several additional noteworthy findings. First, racial-ethnic inequities in community exposure to gun homicides persisted across all cross-classified categories of household poverty status and neighborhood socioeconomic disadvantage. This suggests that gains in income and residential mobility generate increased but unequal protections for Black and Latinx families compared with white counterparts, in part due to additional forms of structural violence and societal oppression over generations [52].

Second, while past research has highlighted the distinctive patterning of community exposure to gun violence by race-ethnicity and income, documenting that Black and Latinx youth in relatively high-income households nonetheless experience gun violence near their homes more often than white youth in poor households [27], our findings point to high rates of past-year exposure for youth residing in high-disadvantage neighborhoods (depending on racial-ethnic subgroup, 50–70%) and relatively low rates of exposure for youth in low-disadvantage neighborhoods (depending on racial-ethnic subgroup, 10–20%), regardless of household income. These findings add to the mounting body of evidence on the importance of place for health and safety and suggest that place-based investments in the social, economic, and structural foundations of community life, particularly in minoritized and marginalized communities, can help reduce the inequitable burden of gun violence, even with limited adjustments to individual-level income. Further supporting such an approach is recent evidence that increases in the number of local nonprofits focused on building stronger communities led to substantial reductions in violence from the 1990s to the 2010s [53].

### Limitations

Our findings should be interpreted in light of several limitations. First, because the FFCWS oversampled unmarried parents at the time of their child’s birth, youth in our study are, on average, socially and economically more disadvantaged than the general population. However, this sampling approach provides an overrepresentation of racially and socioeconomically diverse families who are more likely to be exposed to community gun violence. Additionally, when weighted as in this study, data from the FFCWS are designed to be nationally representative of youth born in 1998–2000 in cities of 200,000 people or more; though, our results may not generalize to youth born in rural areas or smaller cities and towns or to those born in prior or subsequent cohorts, suggesting additional avenues for future research among these populations, as well as studies capable of further disaggregating self-reported race-ethnicity to more fully understand differential experiences of gun violence which otherwise may be masked in aggregate categories such as “other.”

Second, the GVA relies on local and state police, media, data aggregates, government, and other sources to generate a national database of incidents of gun violence, which may result in identification and classification errors. However, a prior evaluation comparing the GVA with statistics from the CDC for the state of Pennsylvania concluded that although the GVA counts of gun homicides were lower than CDC counts, the GVA counts closely track the CDC counts over time, with both secular changes (annual increase in deadly gun violence) and seasonal trends (reduction in deadly gun violence during winter months) consistently reflected in both sources [35]. Further analysis has also found a lack of significant spatial clustering across the USA and only minor differences in the gun-related death rates between the GVA and the CDC [54]. Development and dissemination of a federal reporting system of incident-level violence data would nonetheless be valuable for population research. Furthermore, there may be some measurement error in our estimates of community exposure prevalence if youth moved residences in the year prior to their Year 15 interview; however, past research suggests that moves most often occur between areas characterized by similar social and structural correlates of community violence risk [55].

Third, although the GVA collects information on multiple forms of both fatal and nonfatal gun violence, at this time, only fatal interpersonal gun violence incidents have been linked with the FFCWS. Death is the most severe and irreversible consequence of gun violence and thus provides us with a baseline, likely conservative, understanding of the prevalence of exposure; however, future research will benefit from the inclusion of the full spectrum of both fatal and nonfatal gun violence exposure in youths’ local environments, as well as from the study of other forms of gun violence such as gun suicide, the rate of which has increased among all youth and especially Black youth in recent years.

Finally, this study did not examine the consequences of community exposure to gun homicides among youth. Although beyond the scope of the current objectives, future research should investigate both the average and heterogeneous impacts of community exposure to fatal and nonfatal gun violence, across various distances and time spans, on a range of youth social-emotional health and behavioral outcomes to further inform gun violence prevention and intervention efforts.

## Conclusion

The year 2020 ended with rates of community violence far higher than those seen in recent decades—e.g., homicides increased by 30% and gun assaults by 8% in large US cities compared with 2019—and this trend has persisted through 2021. Much of this violence has most significantly impacted minoritized and marginalized communities, exacerbating longstanding inequities that have been further compounded by the COVID-19 pandemic and the social, psychological, and economic fallout associated with necessary efforts to lessen its spread. As health and social service providers, policymakers, and the public respond to this historic surge, particularly in light of a series of unprecedented commitments from the federal government to invest in community violence intervention strategies, this study provides much-needed and more comprehensive baseline estimates of the prevalence of exposure to gun violence, encompassing not only direct and witnessed gun violence but also gun violence that is experienced in community context. These data are critical for informing prevention and intervention efforts, particularly in communities that are disproportionately impacted by firearm-related harm.

## Appendix

**Table 6:** Distribution of youth across cross-classified categories of household poverty status and neighborhood socioeconomic disadvantage, by race-ethnicity, Fragile Families and Child Wellbeing Study, 2014–2017 (conditional relative prevalence for columns)

		Weighted % (95% *CI*)			
		Household poverty status			
Neighborhood socioeconomic disadvantage		Low (200% + FPL)	Moderate (100–199% FPL)	High (< 100% FPL)	Total
Black	Low	26.07% [18.34, 35.63]	24.24% [11.35, 44.45]	7.84% [4.261, 13.99]	19.56% [13.44, 27.58]
	Moderate	47.25% [33.71, 61.20]	27.34% [14.67, 45.15]	27.03% [20.11, 35.29]	34.00% [27.89, 40.71]
	High	26.68% [16.14, 40.76]	48.42% [28.56, 68.79]	65.13% [55.68, 73.52]	46.44% [39.93, 53.07]
Latinx	Low	41.20% [26.87, 57.19]	15.43% [8.493, 26.38]	11.35% [4.045, 28.02]	25.23% [19.01, 32.67]
	Moderate	44.43% [30.54, 59.24]	52.93% [30.42, 74.30]	34.98% [21.48, 51.42]	44.19% [34.33, 54.53]
	High	14.38% [8.996, 22.20]	31.65% [14.73, 55.38]	53.66% [34.56, 71.75]	30.58% [23.81, 38.32]
White	Low	73.38% [61.39, 82.70]	42.61% [18.59, 70.72]	32.85% [15.05, 57.48]	66.1% [55.10, 75.59]
	Moderate	25.72% [16.52, 37.74]	50.90% [24.01, 77.28]	36.42% [19.21, 57.97]	29.38% [20.64, 39.94]
	High	0.89% [0.11, 6.97]	6.48% [1.91, 19.80]	30.73% [11.96, 59.16]	4.53% [1.91, 10.35]
Other/multi-racial	Low	82.37% [43.75, 96.56]	29.35% [10.72, 58.96]	25.60% [3.78, 75.09]	61.12% [43.68, 76.12]
	Moderate	13.85% [2.118, 54.44]	43.10% [20.02, 69.61]	14.54% [3.60, 43.68]	17.82% [7.16, 37.87]
	High	3.78% [0.79, 16.32]	27.55% [9.23, 58.73]	59.86% [18.19, 90.92]	21.06% [9.71, 39.83]

**Table 7:** Predicted probability of youths’ exposure to gun homicide incidents in the past year within 1300 m of home by race-ethnicity, household (HH) poverty status, and neighborhood (NH) socioeconomic disadvantage, Fragile Families and Child Wellbeing Study, 2014–2017 (*N* = 1991)

HH poverty × NH disadvantage		Coef	SE
Low-low	White	0.10	0.04
	Black	0.22	0.07
	Latinx	0.21	0.06
	Other/multi-racial	0.15	0.06
Low-mod	White	0.22	0.07
	Black	0.40	0.09
	Latinx	0.39	0.09
	Other/multi-racial	0.29	0.10
Low–high	White	0.51	0.11
	Black	0.72	0.06
	Latinx	0.70	0.08
	Other/multi-racial	0.60	0.12
Mod-low	White	0.14	0.06
	Black	0.29	0.08
	Latinx	0.27	0.12
	Other/multi-racial	0.19	0.09
Mod-mod	White	0.28	0.11
	Black	0.49	0.10
	Latinx	0.47	0.16
	Other/multi-racial	0.36	0.14
Mod-high	White	0.59	0.15
	Black	0.78	0.08
	Latinx	0.77	0.13
	Other/multi-racial	0.68	0.16
High-low	White	0.15	0.06
	Black	0.31	0.10
	Latinx	0.29	0.09
	Other/multi-racial	0.21	0.09
High-mod	White	0.30	0.07
	Black	0.51	0.08
	Latinx	0.50	0.09
	Other/multi-racial	0.39	0.11
High-high	White	0.62	0.10
	Black	0.80	0.05
	Latinx	0.79	0.07
	Other/multi-racial	0.70	0.11

**Table 8:** Distribution of youth across cross-classified categories of household poverty status and neighborhood socioeconomic disadvantage, by race-ethnicity, Fragile Families and Child Wellbeing Study, 2014–2017 (relative prevalence for whole table)

		Weighted % (95% *CI*)			
		Household poverty status			
Neighborhood socioeconomic disadvantage		Low (200% + FPL)	Moderate (100–199% FPL)	High (< 100% FPL)	Total
Black	Low	8.86 [5.85, 13.19]	8.17 [3.63, 17.36]	2.54 [1.49, 4.282]	19.56 [13.44, 27.58]
	Moderate	16.05 [10.31, 24.13]	9.21 [4.93, 16.55]	8.74 [5.91, 12.74]	34.00 [27.89, 40.71]
	High	9.07 [5.83, 13.84]	16.31 [10.55, 24.35]	21.06 [13.92, 30.58]	46.44 [39.93, 53.07]
	Total	33.98 [26.48, 42.38]	33.68 [25.93, 42.42]	32.34 [22.87, 43.52]	100
Latinx	Low	17.53 [10.63, 27.53]	4.46 [1.80, 10.62]	3.24 [1.06, 9.457]	25.23 [19.01, 32.67]
	Moderate	18.91 [10.59, 31.45]	15.29 [10.49, 21.75]	9.99 [5.48, 17.52]	44.19 [34.33, 54.53]
	High	6.12 [3.30, 11.06]	9.14 [3.29, 22.94]	15.32 [10.56, 21.72]	30.58 [23.81, 38.32]
	Total	42.56 [27.2, 59.49]	28.89 [18.01, 42.89]	28.55 [21.23, 37.21]	100
White	Low	58.40 [46.75, 69.17]	4.33 [1.99, 9.14]	3.38 [1.72, 6.51]	66.1 [55.1, 75.59]
	Moderate	20.47 [13.45, 29.88]	5.168 [2.17, 11.81]	3.74 [1.92, 7.16]	29.38 [20.64, 39.94]
	High	0.71 [0.09, 5.41]	0.66 [0.19, 2.24]	3.16 [1.09, 8.83]	4.53 [1.91, 10.35]
	Total	79.57 [70.76, 86.25]	10.15 [5.74, 17.35]	10.27 [6.68, 15.47]	100
Other/multi-racial	Low	50.84 [32.76, 68.7]	3.81 [1.38, 10.07]	6.48 [1.343, 26.07]	61.12 [43.68, 76.12]
	Moderate	8.55 [1.266, 40.54]	5.59 [2.19, 13.53]	3.68 [1.32, 9.836]	17.82 [7.16, 37.87]
	High	2.33 [0.48, 10.57]	3.57 [1.26, 9.705]	15.16 [5.127, 37.12]	21.06 [9.71, 39.83]
	Total	61.72 [44.3, 76.57]	12.97 [6.532, 24.1]	25.32 [12.42, 44.75]	100

## Footnotes

1 We refer to fatal gun violence incidents as gun homicides. However, the fatal gun violence incidents linked to the FFCWS may include unintentional and undetermined firearm deaths, which nationally, made up approximately 2.1% of annual deaths from firearms, on average, during the Year 15 data collection period, 2014-2017.
